# Down-regulation of Notch signaling during corneal epithelial proliferation

**Published:** 2008-06-05

**Authors:** A.R. Djalilian, A. Namavari, A. Ito, S. Balali, A. Afshar, R.M. Lavker, B.Y. J. T. Yue

**Affiliations:** 1 Department of Ophthalmology, University of Illinois at Chicago, Chicago, IL; 2 Department of Dermatology, Northwestern University Feinberg School of Medicine, Chicago, IL

## Abstract

**Purpose:**

We evaluated the expression and activation of Notch pathway genes in the adult human and murine corneal epithelium during proliferation.

**Methods:**

The expression of Notch pathway genes in the limbal and central human corneal epithelium was compared by reverse transcription polymerase chain reaction (RT–PCR). Their expression pattern was examined by immunofluorescence and in situ hybridization. The temporal expression of *Notch1* during murine wound healing was assessed by RT–PCR. Notch activity was determined using western blot for the Notch intracellular domain (NotchIC). The expression of *Hes1* was evaluated in cell culture.

**Results:**

The expression of *Notch1* and *Jagged1* was higher in the human limbal epithelium while the expression of *Hes1* and *Hes5* was higher in the central cornea. Expression of *Notch1*, *Jagged1*, and *Hes1* was found predominantly in the basal and immediate suprabasal cells. During neonatal corneal development, NotchIC was detected in occasional cells at P10 while at P15 and P90, it was found in the basal and early suprabasal layers. NotchIC was found to be lower in the limbal compared to central corneal epithelium. The expression of *Notch1* was lower at 24 h post-wounding but was completely restored in six days. The levels of NotchIC were decreased at 24 h post-wounding and after application of topical phorbol myristate. In vitro, the expression of *Hes1* was higher in confluent cells maintained under high calcium conditions.

**Conclusions:**

The inverse correlation between Notch signaling and the proliferative status of the corneal epithelium is consistent with the idea that Notch plays a role in corneal epithelial differentiation.

## Introduction

The cornea is covered by a stratified squamous epithelium that functions to maintain transparency as well as provide the first line of defense against pathogens and the penetration of noxious agents. The corneal epithelium is continually losing cells due to desquamation (primarily via the blink reflex) and thus must replace these cells to maintain homeostasis. Such continuously renewing epithelia are by definition governed by stem cells. The stem cells of the corneal epithelium are preferentially located in the limbal region [1]. Upon division, the slow cycling limbal epithelial stem cells give rise to daughter cells known as transient amplifying (TA) cells. The TA cells in turn undergo multiple cell divisions giving rise to more differentiated epithelial cells that migrate centrally and leave the basal layer toward the superficial corneal epithelium [2,3]. This highly organized differentiation program is under the control of multiple regulatory mechanisms, which remain largely unknown [4,5].

The Notch signaling pathway plays a key role in the proliferation and differentiation of many tissues. It is a highly conserved network that orchestrates cell-fate decisions in the nervous, gastrointestinal, and the hematopoietic systems of many organisms ranging from insects to humans. Notch proteins are large transmembrane proteins. Four Notch genes have been identified in mammals (*Notch1*-*Notch4*), and their mutations cause severe abnormalities in organ development and adult homeostasis [6]. In addition, three Delta (Delta like 1, 3, and 4) and two Jagged (*Jagged1* and *Jagged2*) proteins have been identified as Notch ligands in mammals. Notch signaling is initiated by the interaction between the ligand on one cell and the receptor on a neighboring cell. This interaction triggers the proteolytic cleavage of the Notch receptor at the membrane proximal region by the γ-secretase presenilin [7,8]. In the most well characterized “canonical” pathway in mammalian cells, the Notch intracellular domain (NotchIC) translocates to the nucleus where it mostly associates with the recombination signal binding protein for the immunoglobulin kappa J region (RBPJκ). The NotchIC/RBPJκ complex transactivates protein targets such as Hairy/Enhancer of Split (*HES*) and Hairy/Enhancer of Split-related with YRPW motif (*HEY*) genes, which in turn affect numerous pathways involving cell-fate determination [9].

The role of Notch signaling in the corneal epithelium has not been studied in detail. Its importance in corneal epithelial proliferation and differentiation was initially discovered while studying *Notch1* in the mouse epidermis. When *Notch1* was conditionally deleted in the epidermis and the corneal epithelium of postnatal mice, hyperproliferation and abnormal differentiation were noted in the epidermis and the corneal epithelium [10]. In the present study, we confirm and extend further the role of Notch signaling in the adult corneal epithelium by demonstrating that Notch activity is down-regulated in the corneal epithelium during the early phase of proliferative conditions.

## Methods

### Immunostaining

Three human corneas (obtained from the Illinois Eye Bank) or mouse whole eyes were embedded in OCT and then cut into 8 μm sections. The sections were fixed for 10 min in chilled acetone and blocked with 5% donkey serum for 1 h at room temperature. The sections were incubated for 90 min at room temperature with rat monoclonal anti-Notch1 antibody (Developmental Studies Hybridoma Bank, Iowa City, IA), rabbit polyclonal anti-Notch 1 (1:100) or goat polyclonal anti-Jagged1 (1:100; both from Santa Cruz Biotechnology, Santa Cruz, CA) or a rabbit polyclonal anti-cleaved Notch-1 (1:200; Cell Signaling Technology, Danvers, MA or Abcam, Cambridge, MA) or rabbit monoclonal anti-Ki67 (1:250; Labvision, Fremont, CA). For a negative control, the sections were incubated with an irrelevant (no epithelial expression of the antigen) rabbit or goat antibody. A fluorescein-conjugated donkey anti-rabbit (1:200) or donkey anti-goat (1:200) was used for 1 h at room temperature. The slides were counterstained with DAPI or propidium iodide, visualized using a Zeiss Axiovert fluorescence microscope, and photographed with an AxioCam (Carl Zeiss, Thornwood, NY) camera. For immunohistochemistry, a similar protocol was followed except a biotin-conjugated goat anti-rabbit antibody (1:500) was used, and sections were incubated in streptavidin peroxidase complex before being developed in 3,3′ diaminobenzadine (all from Vector Laboratories, Burlingame, CA).

### Relative quantitative reverse transcription polymerase chain reaction

Epithelial cells from the central corneal and limbal regions were separately removed from three fresh eye bank corneas (donor ages 41, 56, and 62). Specifically, a 7.5 mm trephine was used to excise the central cornea leaving a 1.5 mm area of limbal epithelium intact. The central button and the limbal rims were placed in dispase (5 mg/ml) for 1 h at 37 °C, and the epithelium was gently separated from the underlying stroma by blunt dissection and stored in RNAlater (Qiagen, Valencia, CA). For the animal experiments, corneas that were removed following euthanasia were similarly stored in RNAlater.

Total RNA was isolated from the human epithelial cells (limbal and central) or the mouse corneal epithelium per manufacturer’s instructions (RNeasy Protect Mini Kit, Qiagen). Reverse transcription was performed with 0.5 µg total RNA using random hexamers and a cDNA synthesis kit (SuperScript First-Strand Synthesis System, Invitrogen, Carlsbad, CA). Relative quantitative polymerase chain reaction (PCR) was performed according to the manufacturer’s instructions (QuantumRNA Universal kit; Ambion, Austin, TX) using primer sets for 18S rRNA as an internal standard and intron-spanning primers for human or mouse *Notch1*, *Notch2*, *Delta1*, *Jagged1*, *Jagged2*, *Hes1*, *Hes5*, *DeltaN-p63*, and *ABCG2*. The linear range of the PCR for each target gene was determined experimentally using cDNA from the control sample. The cycle number in the middle of the linear range of the PCR was chosen for each specific target gene. Following the manufacturer’s instructions, the primers for the 18S rRNA were mixed with varying ratios of competimers (ranging from 1:9 to 4:6) to find the optimal primer:competimer ratio, which provided near equal bands for 18S and the target gene. For each sample, a multiplex PCR for both the target gene and 18S internal standard was then performed and the products were visualized on 1.2% agarose gels after ethidium bromide staining. All samples were run in duplicates and repeated a minimum of three times. For every primer set, a sample with no reverse transcriptase was used as the negative control.

The band densities from each reaction were quantified (Digital Science Image Station, model 440CF; Eastman Kodak, Rochester, NY) and subsequently normalized by dividing the band density of the target gene by the density of its corresponding 18S band. The normalized densities were converted into a ratio between the central cornea and the limbus and reported as means±standard deviation. Statistical comparison was performed using Student’s *t*-test and a p value less than 0.05 was considered significant.

### In situ hybridization

In situ hybridization was performed on human corneal paraffin sections [11]. Both the sense (negative control) and anti-sense probes were digoxigenin-labeled RNA probes. The RNA probes were synthesized by in vitro transcription (Riboprobe In Vitro Transcription System, Promega, Madison, WI) using T7 RNA polymerase, digoxigenin labeled UTP (Roche, Nutley, NJ), and a template amplified from cDNA by PCR. The template was a 624-bp region in the UTR of *Hes1* and was amplified such that the sequence for the T7 bacteriophage promoter was built into the reverse primer sequence (Forward: 5′-ATC AAT GCC ATG ACC TAC CC-3′, Reverse: 5′-CTA ATA CGA CTC ACT ATA GGG AGG CGC AAT CCA ATA TGA AC-3′). The T7 promoter sequence was built into the forward primer for sense probe synthesis.

The sections were deparaffinized, digested with proteinase K (10 µg/µl), and post-fixed in 1% paraformaldehyde. After pre-hybridization, the sections were incubated with the pre-heated Hes1 probe (diluted in hybridization solution) overnight at 50 °C and sequentially washed with serial dilutions of sodium chloride sodium citrate (SSC) buffer. The sections were incubated at room temperature with blocking solution for 1.5 h followed by incubation with 1:500 alkaline phosphatase-conjugated anti-digoxigenin antibody at room temperature. The slides were washed and incubated further with nitroblue tetrazolium/ 5-bromo-4-chloro-3-indolyl-phosphate chromogen (NBT/BCIP) for 30–120 min at room temperature in the dark and then rinsed and mounted with Vectashield (Vector Laboratories, Burlingame, CA). Positive reaction products appeared purplish blue.

### Mouse model of corneal epithelial wound healing

A mouse model of corneal epithelial wound healing was used to study the expression of Notch pathway components under proliferative conditions [12]. Six-month-old C57Bl/6 mice were anesthetized with an intraperitoneal injection of ketamine (100 mg/kg) and xylazine (5 mg/kg). After applying several drops of topical 0.5% proparacaine, a 1.5-mm area of the central epithelium was demarcated and removed by gentle scraping (excluding the limbal area). At 24-h, 48-h, and144-h post-wounding time points, the corneal epithelium was removed by scraping (different mice used for each time point). Between 8 and 10 corneal epithelial scrapings were pooled together for each time point. Total RNA was extracted from the samples and subjected to relative quantitative RT–PCR with 18S rRNA as an internal standard (Ambion).

### Pharmacologic induction of corneal epithelial proliferation in vivo

To stimulate proliferation of the ocular surface epithelial cells, the tumor promoting agent phorbol myristate ester (TPA) was dissolved in pertolatum at a concentration of 0.5%. This concentration has been demonstrated to induce a hyperproliferative response that maximizes after 24 h with the absence of necrosis [13]. Six-month-old C57BL/6 mice received topical 0.5% TPA in petrolatum while the control mice received petrolatum alone. Corneas were harvested after 24 h and prepared for western blot analysis.

### Western blot

Following homogenization, the mouse corneal proteins were extracted overnight with radioimmunoprecipitation assay (RIPA) buffer supplemented with protease inhibitors. Equal amounts of each sample were mixed with sample buffer (Invitrogen), denatured by heating at 95 °C for 10 min, and subjected to electrophoresis on 10%–20% Tricine gels (Invitrogen). The protein bands were transferred to nitrocellulose membranes, and equal loading was verified by Ponceau S staining. The membranes were incubated in 3% BSA in Tris-buffered saline for 1 h followed by an overnight incubation (4 °C) with rabbit anti-NotchIC antibody (1:1000; Cell Signaling). The membranes were washed with Tris-buffered saline with 0.05% Tween 20 and incubated with HRP-goat-anti rabbit IgG (1:5000) for 1 h at room temperature. Detection was performed with enhanced chemiluminescence reagents (Pierce Biotechnology, Rockford, IL) and recorded on film (Kodak X-OMAT; Eastman Kodak Co., Rochester, NY).

### Cell culture

Human corneal epithelial cultures were initiated from corneoscleral rims obtained from eye bank eyes. The rims were treated with dispase (10 mg/ml) at 4 °C overnight to disrupt the basement membrane. The epithelial sheets were peeled off and digested in 0.25% trypsin-EDTA at 37 °C for 5–10 min. Cells were washed and resuspended in a keratinocyte serum-free medium (KSFM; Invitrogen) and plated in tissue culture plates. After the first passage, the cells were plated at either low density (20%–30% confluence) where proliferation is induced or high density cultures (>90% confluence) where proliferation is inhibited. To induce differentiation, the low density cultures were exposed to 1.6 mM calcium. At 48 h, the cultured epithelial cells were trypsinized and subjected to relative quantitative RT–PCR with normalization to 18S.

## Results

### Expression of Notch pathway genes in the human limbal and central corneal epithelium

Human corneal epithelial cells isolated from fresh eye bank eyes were initially screened for the presence or absence of Notch pathway genes by RT–PCR. Expression of *Notch1, Notch2*, *Delta1*, *Jagged1*, *Jagged2*, *Hes1*, and *Hes5* was observed with all transcripts detected at 28 cycles or less. The transcripts for *Delta4*, *Hes2*, *Hey1, Hey 2*, and *HeyL* were detected only after amplification for 35–40 cycles. These genes were considered to be of very low abundance, and the possibility of non-epithelial origin of their expression (e.g., resident immune cells) could not be excluded. The expression of *Notch3*, *Notch4*, *Delta3*, and *Hes3* was not detected in the human corneal epithelium.

**Figure 1:**
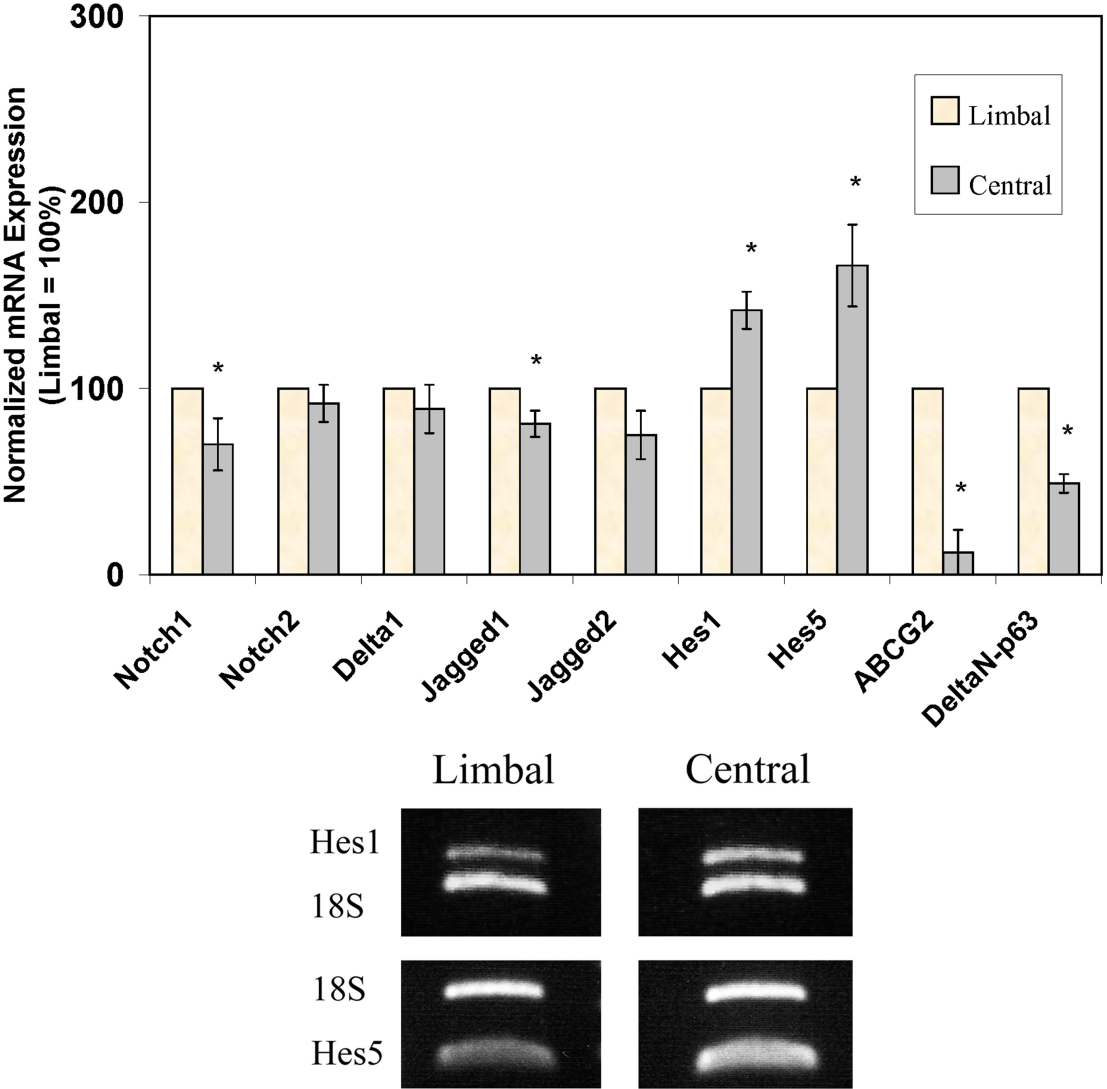
Relative quantitative reverse transcription polymerase chain reaction comparing the expression of Notch pathway genes between the limbal and central human corneal epithelium. All results are after normalization to 18S rRNA. Higher expression in the central cornea is noted for *Hes1* (1.4 fold) and *Hes5* (1.7 fold). The expression of *Notch1* (0.7 fold) and *Jagged1* (0.8 fold) was lower in the central cornea compared to the limbus. The expression of *Jagged2*, *Notch2*, and *Delta1* appeared slightly lower in the central cornea, but this did not reach statistical significance (*p<0.05). As a positive control, the expression of limbal markers, *DeltaNp63* and *ABCG2*, was markedly higher in the limbus than the central cornea. Representative gels of the relative quantitative RT–PCR for *Hes1* and *Hes5* are shown.

Using relative quantitative RT–PCR, the expression of the Notch pathways genes was compared between the limbal and the central corneal epithelium. The limbal expression of Notch1 receptor and Jagged1 ligand was respectively 30%±14% and 19%±7% fold lower in the central cornea. On the other hand, the expression of the canonical Notch downstream targets, Hes1 and Hes5, were 42%±10% and 66%±22% higher, respectively, in the central cornea compared to the limbus (p<0.05). There was no statistical difference in the level of expression of *Notch2*, *Delta1*, and *Jagged2* between the limbal and central corneal epithelium. As a positive control, the expression of limbal markers, DeltaN-p63 and ATP-binding cassette transporter isoform G2 (ABCG2), were both markedly higher in the limbus as previously described [14,15]. Overall, these results demonstrate that while the Notch1 receptor is expressed at a higher level in the limbus, the level of Notch activity as measured by the expression of its downstream targets, Hes1 and Hes5, is higher in the central corneal epithelium (Figure 1).

**Figure 2:**
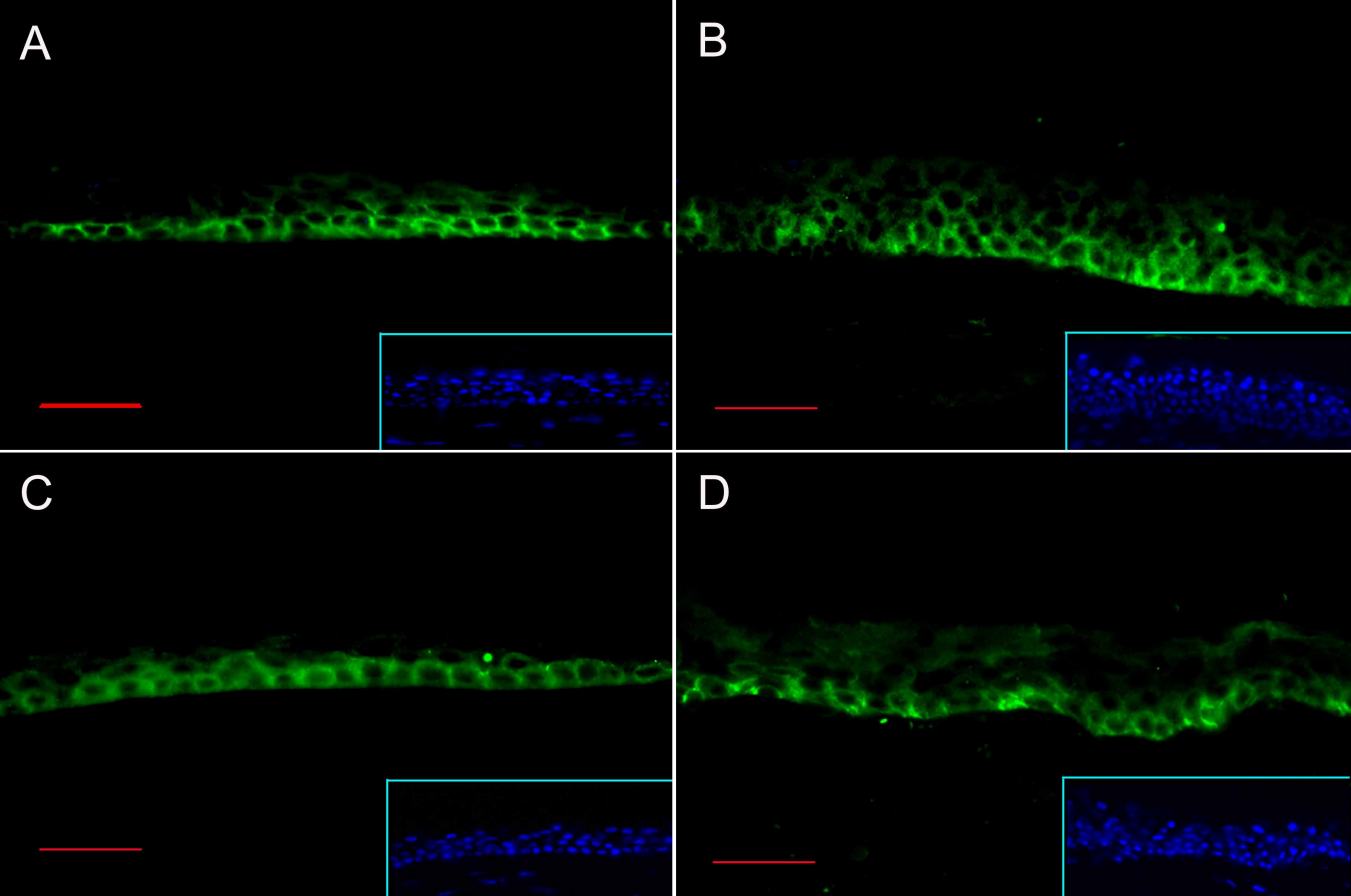
Immunofluorescence staining for Notch1 and Jagged1. Notch1 (**A** and **B**) and Jagged1 (**C** and **D**) staining is noted in the basal and immediate suprabasal layers of central human cornea (**A** and **C**) and limbus (**B** and **D**). Inset show the nuclear staining of the sections with DAPI. Bar=30 μm.

By immunofluorescence, both Notch1 and Jagged1 were found to be expressed predominantly in the basal and suprabasal layers of the human corneal epithelium (Figure 2A,C). Notch1-stained cells extended higher in the limbal epithelium (Figure 2B) than in the corneal epithelium (Figure 2A).

**Figure 3:**
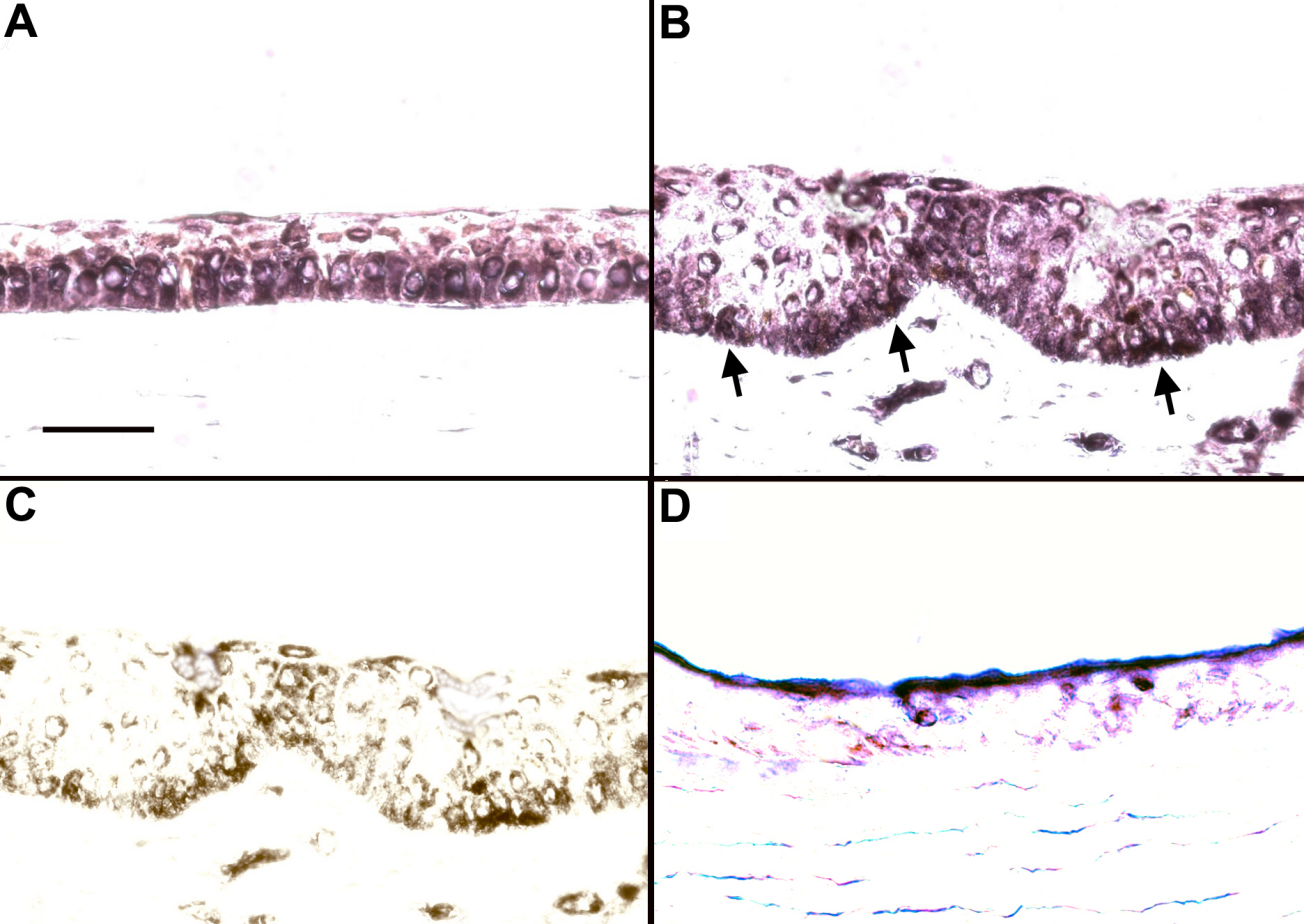
In situ hybridization of the human *Hes 1* gene. In both the central corneal epithelium (**A**) and the limbal epithelium (**B**), expression of *Hes1* is seen predominantly in the basal layer with some extension into the suprabasal layers. The intensity of the staining was slightly lower in the limbus. The areas with darker staining observed in the limbal basal epithelium (arrows) actually represent underlying pigmentation of the tissue. The pigmented cells in the limbus are seen more clearly in **C**, which represents the same section in **B** without the purple staining. Minimal non-specific staining is observed in the sense control in the central cornea (**D**). Bar=30 μm.

The mRNA expression pattern of *Hes1*, a downstream target of Notch, was evaluated by in situ hybridization. Its expression was localized to the basal and immediate suprabasal layers of the corneal epithelium (Figure 3A). In comparison, a more patchy expression pattern was detected in the limbal epithelium (Figure 3B). Overall, the expression pattern of *Hes1*, which indicates Notch activation in the basal and suprabasal cell layers, was consistent with the immunofluorescent expression patterns of Notch1 and Jagged1.

### Activation of Notch in the mouse corneal epithelium during development and during proliferative states

**Figure 4:**
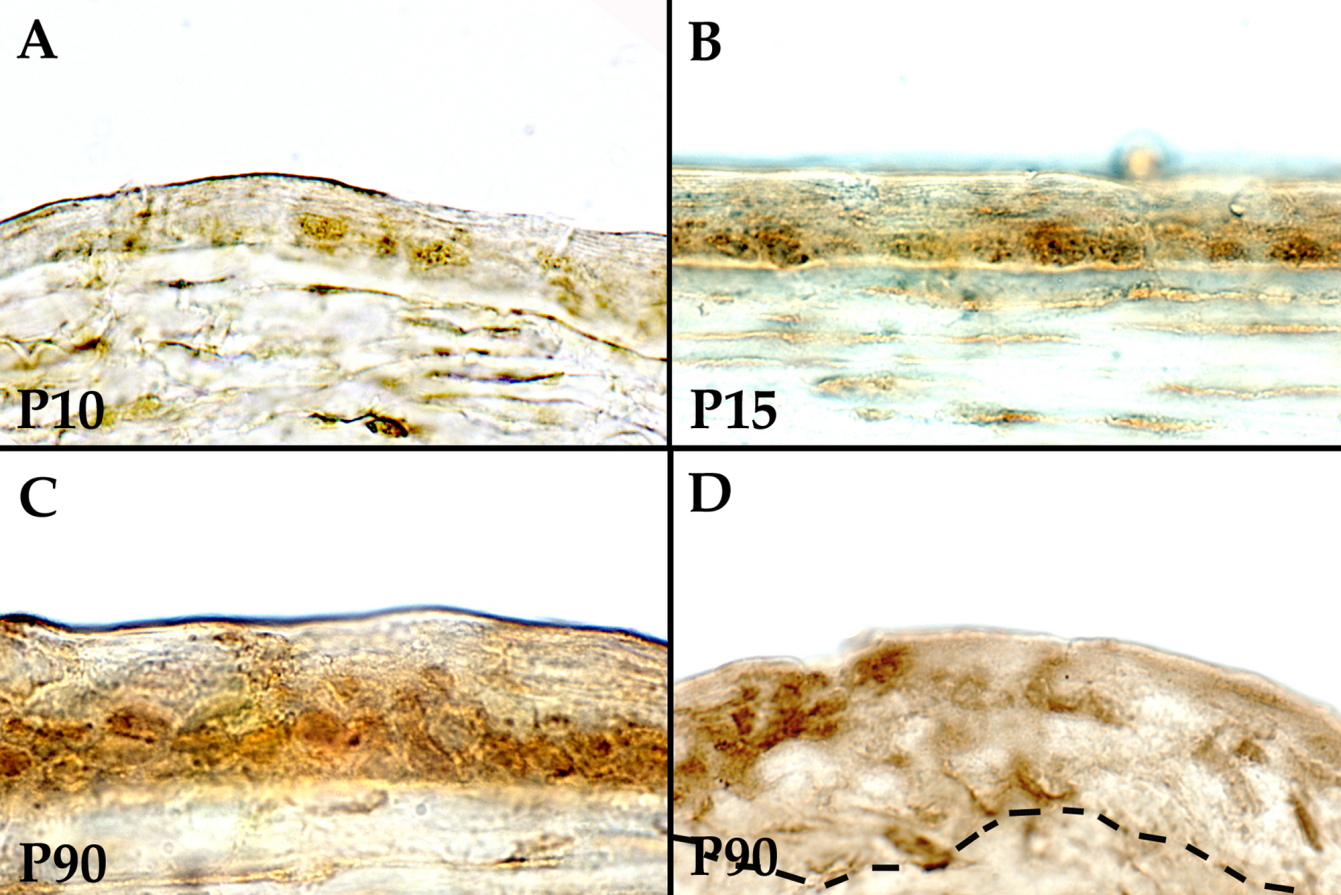
Immunohistochemical staining for NotchIC, the cleaved Notch intracellular fragment in developing mouse corneas. **A**: At P10, weak NotchIC staining is found in occasional basal cell of the corneal epithelium. **B**: By P15, NotchIC is present in many of the basal cells. (**C**) At P90, NotchIC is found throughout the basal and immediate suprabasal layers of the mouse corneal epithelium. **D**: In the limbal epithelium at P90, NotchIC is seen in few cells, mostly in suprabasal cells. The area closest to the central cornea (left side of the tissue) demonstrates more staining. Incidentally, NotchIC staining is also noted in the keratocytes (**A**-**D**). Bar=30 μm.

During mouse corneal development, the rate of proliferation as measured by the labeling index increases steadily in the first week of life and peaks at around P7-P10. The rate of proliferation then begins to decline rapidly before eye opening around P12. By P15, the proliferation is approaching the same level typically seen in adult corneal epithelium [16]. Therefore, Notch activation in the mouse cornea was evaluated during these periods by immunohistochemistry. At P10, NotchIC was detected in occasional basal or suprabasal cells. By P15, NotchIC was detected in most of the basal cells, and by P90, it was detectable in all of the basal and early suprabasal cells of the corneal epithelium (Figure 4A-C). At P90, NotchIC staining was seen mostly in the suprabasal cells and very few basal cells in the limbal epithelium (Figure 4D). In general, the limbal epithelium demonstrated less NotchIC compared to the central cornea. Therefore, it appears that in the developing mouse corneal epithelium, Notch activity is inversely correlated with the degree of proliferation.

**Figure 5:**
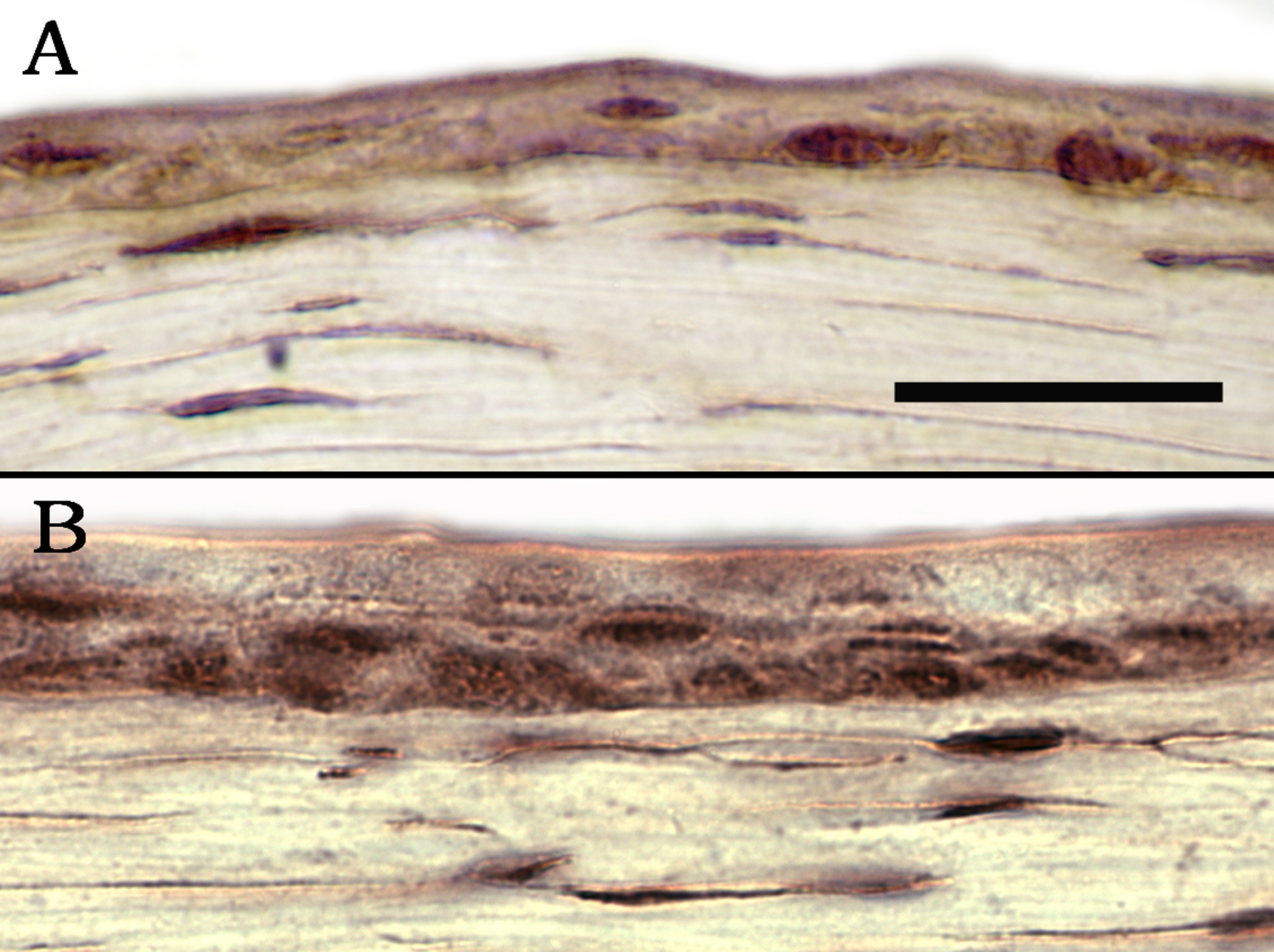
Immunohistochemical staining for NotchIC in the cornea of a wounding mouse model. **A**: NotchIC is seen in just a few basal and some suprabasal cells 24 h after wounding. **B**: In the unwounded control mouse cornea, NotchIC staining is demonstrated in the basal and suprabasal cells of the corneal epithelium. The NotchIC staining is stronger in the unwounded cornea (compare **B** versus **A**). Bar=30 μm.

During wound healing, the corneal epithelial cells that were peripheral to the wounded area have been shown to undergo proliferation [17]. The level of Notch activation in the peripheral cornea was therefore evaluated following a central 1.5-mm epithelial wound. At 24 h, NotchIC staining was found to be reduced in the corneal epithelium that was peripheral to the wounded area (Figure 5A) compared to the same region in a non-wounded mouse cornea (Figure 5B).

**Figure 6:**
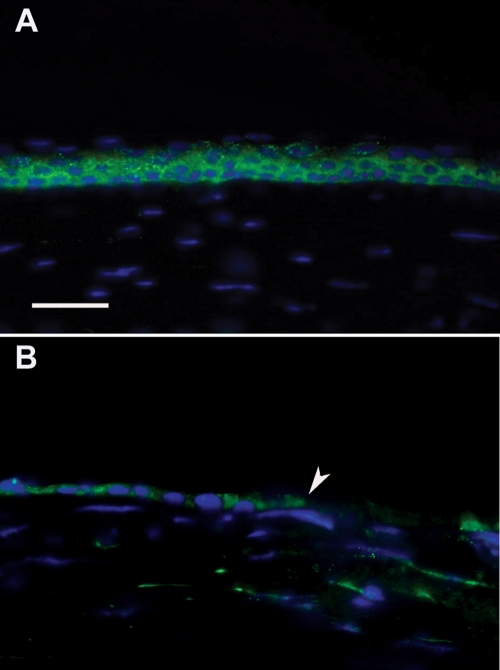
Immunofluorescence for Notch1 in the cornea of a wounding mouse model. **A**: Notch1 expression is noted predominantly in the basal and early suprabasal cells in unwounded mouse corneas. **B**: Scattered expression of Notch1 is seen in the new epithelium near the leading edge (arrowhead) 24 h after wounding. Nuclei were stained with DAPI in blue. Bar=30 μm.

The expression pattern of Notch1 in the adult mouse corneal epithelium was examined by immunofluorescence (Figure 6A). Under normal conditions, Notch1 was found to be expressed in the basal and immediate suprabasal cells of the mouse corneal epithelium, similar to what was observed in human corneas. Notch1 was expressed in the epithelial cells at the leading edge, albeit, at reduced levels 24 h after wounding (Figure 6B).

**Figure 7:**
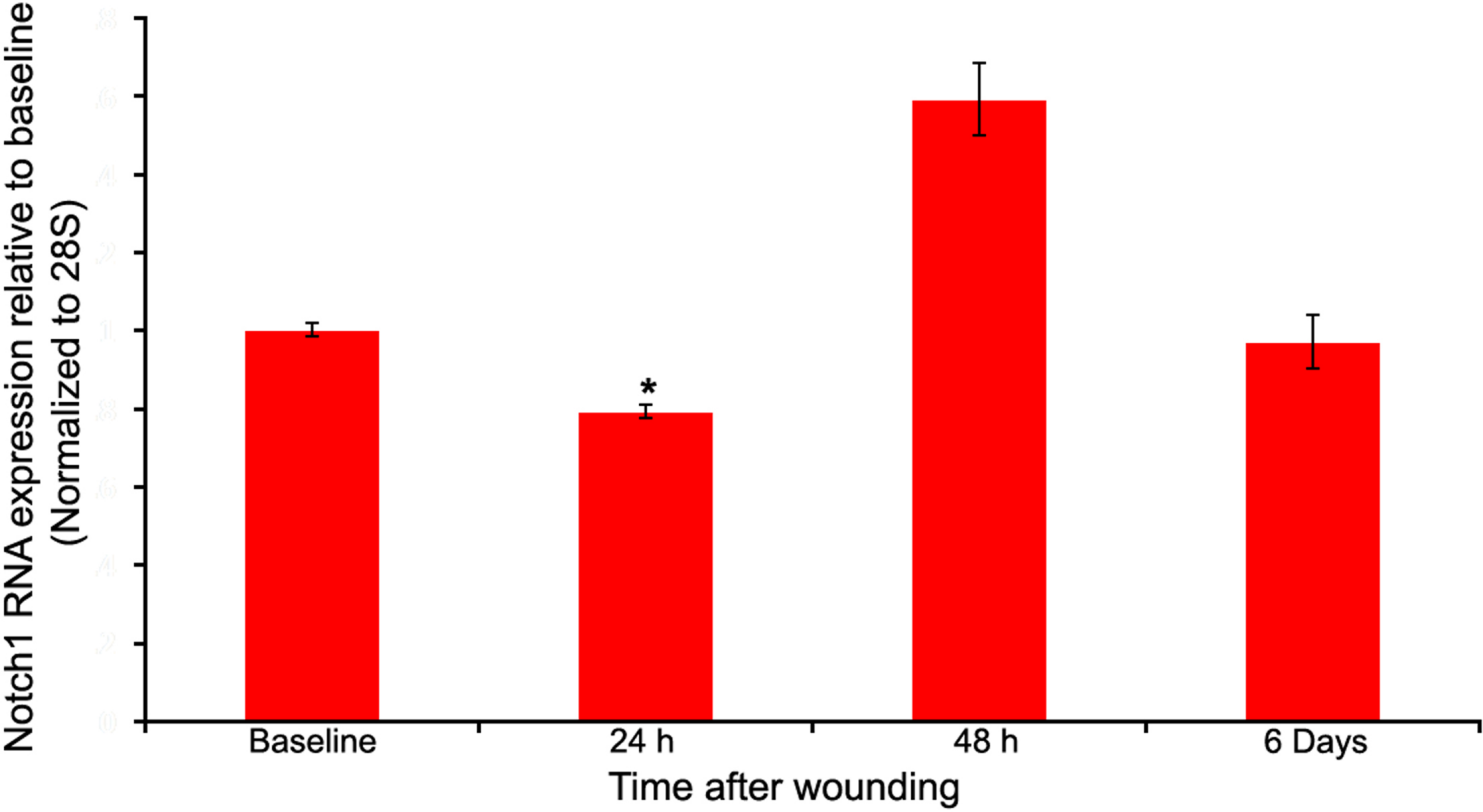
Expression of Notch1 in the mouse corneal epithelium after wounding as determined by relative quantitative reverse transcription polymerase chain reaction. The expression levels are presented relative to the baseline unwounded samples. When the rate of proliferation had peaked at 24 h, Notch1 expression was reduced (78%±3%, p<0.001). By 48 h, the expression had recovered and increased beyond the baseline. After six days, Notch1 returned to baseline levels.

The mRNA expression of *Notch1* was evaluated 24 h, 48 h, and 144 h after wounding by relative quantitative RT–PCR. The results indicated down-regulation of the Notch1 receptor at 24 h following injury (p<0.001). The expression level increased beyond the baseline at 48 h and was back to baseline in six days (Figure 7).

**Figure 8:**
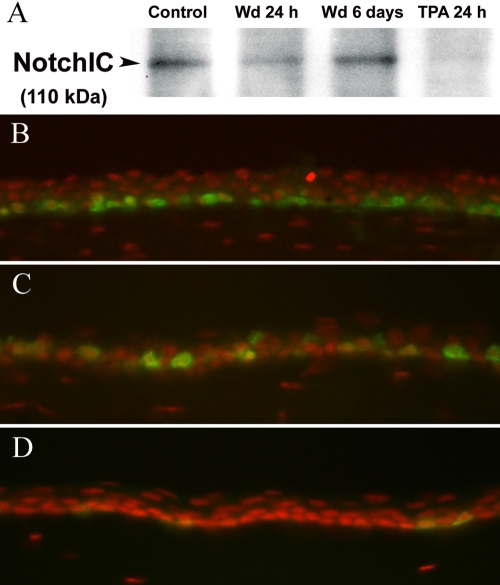
Western blotting for the cleaved Notch intracellular domain. **A**: The results demonstrate reduced levels of Notch intracellular domain (NotchIC) at 24 h after wounding (Wd) and after TPA treatment. Immunofluorescence staining for Ki67 (green) demonstrates the number of proliferating basal cells to be highest in the TPA-treated cornea at 24 h (**B**) followed by the wounded cornea at 24 h (**C**) and a normal unwounded cornea (**D**). The nuclei are stained red with propidium iodide.

To quantify Notch activation at the protein level, the levels of the active intracellular domain of Notch (NotchIC) was compared by western blot analysis. At 24 h post-wounding and 24 h after TPA treatment, the levels of NotchIC was significantly suppressed, indicating down-regulation of Notch activity during these hyperproliferative states (Figure 8A). The increased proliferation in the cornea epithelium was confirmed by Ki67 staining. As expected, the TPA-treated corneal epithelium demonstrated the highest level of proliferation followed by the wounded corneal epithelium and the normal mouse cornea (Figure 8B-D).

### Expression of Hes1 in human corneal epithelial cells in vitro

**Figure 9:**
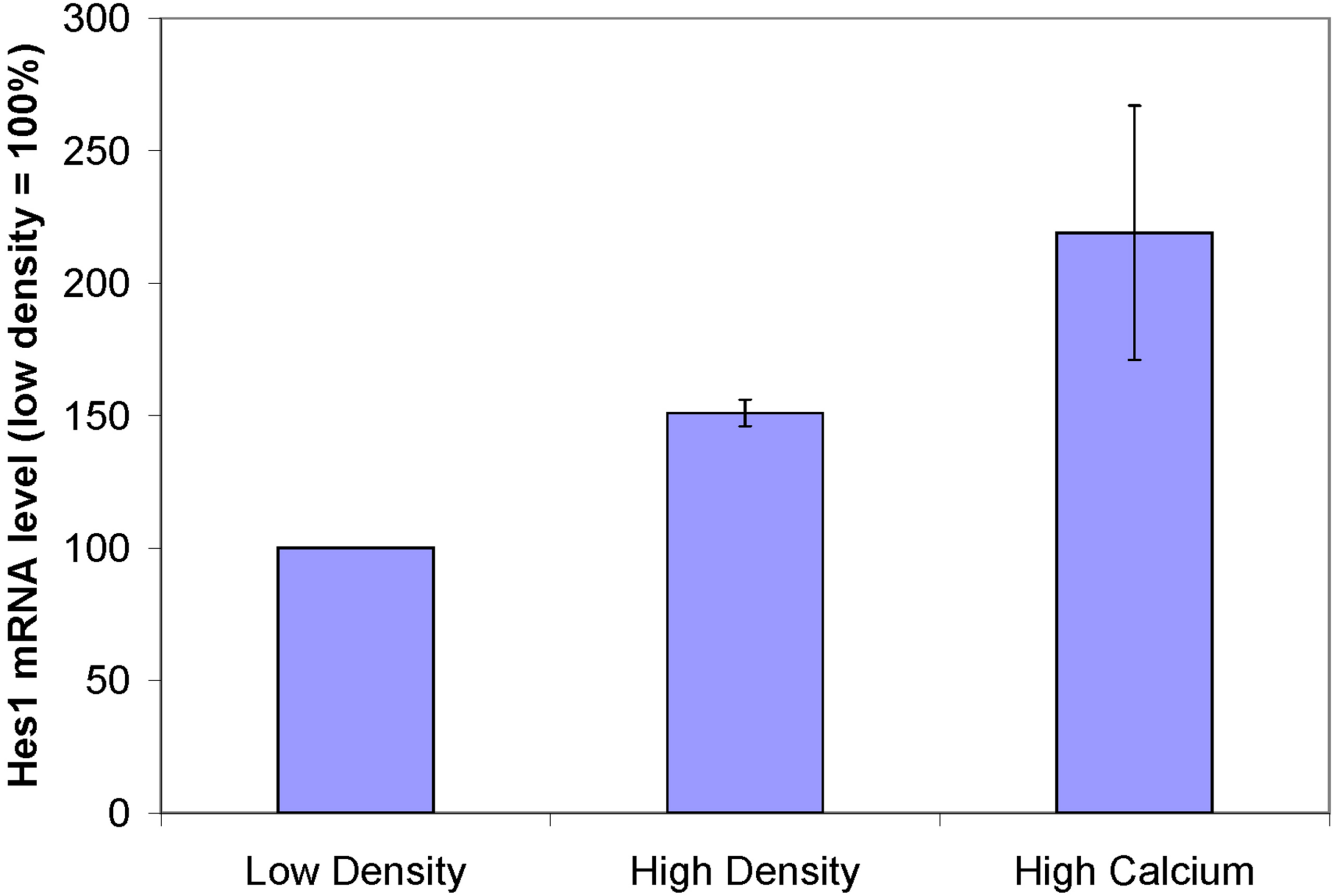
Expression of *Hes1* in corneal epithelial cultures. The level of canonical Notch activity in human corneal epithelial cell cultures was determined by relative quantitative RT–PCR of the downstream target, *Hes1*. After normalization to 18S, the level of *Hes1* in the low density cultures (20%–30% confluence) was arbitrarily set at 100%. High density cultures demonstrated a 1.5 fold increase (151%±5%, p<0.05) and high calcium cultures expressed a 2.2 fold (219%±48%, p<0.05) increase in the level of *Hes1* relative to the low density cultures.

The level of canonical Notch activity as measured by the expression of Hes1 was examined under in vitro conditions. Primary human corneal epithelial cells after the first passage were cultured at high or low densities and high or low (0.09 mM) calcium. The expression of Hes1 was 1.5 fold higher in high density compared to low density cultures (p<0.05). Increasing the calcium concentration to 1.6 mM, which induces differentiation of the corneal epithelial cells, likewise enhanced the expression by twofold (p<0.05). These results demonstrate that the level of Notch activity as measured by the expression of Hes1 is higher in conditions where there is less proliferation and a greater commitment toward differentiation. These results corroborate the in vivo findings that Notch activity is down-regulated in response to hyperproliferation (Figure 9).

## Discussion

Notch signaling is a fundamental pathway that controls cell fate decisions during development and differentiation in nearly all tissues. In the epidermis, a structure which is morphologically similar to the corneal epithelium, Notch signaling functions as a commitment switch that signals the epithelial cells to leave the basal layer and begin terminal differentiation [18,19]. In the murine epidermis, Notch signaling was shown to limit proliferation by inhibiting DeltaNp63α, a cell cycle regulator important for maintaining the proliferative capacity of epidermal progenitor cells [20]. Inhibition of Notch signaling was found to suppress keratinocyte commitment to differentiation and expand populations with stem cell potential [18]. Clinically, Notch is demonstrated to be down-regulated in hyperproliferative conditions such as psoriasis and squamous and basal cell carcinomas [18,21]. Likewise, in animal models, Notch expression is reduced in the first 24 h after wounding of the skin [22].

The importance of Notch signaling in the corneal epithelium was first noted in a study involving the conditional knockout of *Notch1* in the postnatal mouse epidermis [10]. When *Notch1* was conditionally deleted using the basal corneal epithelium-active keratin14 or keratin5 promoter, the mice developed hyperplastic, keratinized, skin-like epithelium in the cornea [10]. This phenotype was particularly manifested after repeated corneal epithelial scraping [23]. Mechanistically, *Notch1* was shown to regulate vitamin A metabolism in the corneal epithelium by regulating the expression of cellular retinol binding protein 1 [23]. These results are confirmed and further extended by our current study where we found Notch activity to be down-regulated during proliferative states in the corneal epithelium. We surmise that Notch signaling must first be down-regulated during the initial stages of wound healing to allow epithelial cells to revert to a more basal and undifferentiated “wound healing” phenotype. Later, when the epithelial defect has been covered by a provisional epithelium, Notch signaling must be restored for the proper differentiation of the corneal epithelial cells to take place. Thus, in the absence of Notch1, the epithelial cells remain in an undifferentiated basal phenotype, which by default follow an epidermal fate.

In this study, we found Notch to be more active in the central corneal epithelium than in the limbus. The level of Notch activation as judged by the expression of its downstream targets, Hes1 and *Hes5*, was 1.42 and 1.66 fold higher in the human central corneal epithelium compared to the limbal epithelium. The result for Hes1 is consistent with that from a previous study in which serial analysis of gene expression found *Hes1* expression in the rat central epithelium to be 1.33 fold higher than in the limbal epithelium [24]. Overall, this is consistent with the fact that the central corneal epithelium has less proliferative capacity and more commitment toward differentiation compared to the limbus [3].

The expression pattern of *Notch1*, *Jagged1*, and *Hes1* in the basal and immediate suprabasal cells further supports the involvement of Notch in the commitment of a cell to proliferate or differentiate. The expression of Notch1 receptor being primarily in the basal and early suprabasal cells of mouse and human corneal epithelium with a slightly higher expression in the limbal epithelium compared to the central cornea is consistent with other reports using fresh tissue or freshly isolated population of cells [23,25,26]. In contrast, *Notch1* expression has been shown to be limited to the corneal suprabasal cells with minimal expression in the corneal basal cells and the limbal epithelium [27]. This difference is most likely due to the choice of primary antibodies. In the present study, we used a rat monoclonal antibody to detect the Notch1 receptor in a manner similar to that of Thomas et al. [26] while Ma et al. [27] used a polyclonal rabbit antibody. Using a different antibody against NotchIC, the active form of Notch, both our laboratory and Vauclair et al. [23] found Notch to be active in the basal and suprabasal cells of murine corneal epithelium. It is important to recognize that while Notch receptors and ligands are present both in the limbal and the central corneal epithelium, the actual level of Notch activity appears to be greater in the central corneal epithelium. This is evident by the higher expression of downstream Hes factors in the central cornea and by the phenotype of the *Notch1* conditional knockouts where the central cornea develops keratinization while the limbus is relatively spared [10,23].

It is interesting to note that the expression pattern of Notch1 and Hes1 in the corneal epithelium is slightly different from the epidermis where their expression is primarily suprabasal. This may reflect inherent differences in the architecture of these tissues. In particular, as previous studies have suggested, the basal cells in the peripheral and central corneal epithelia behave more like the suprabasal cells in the epidermis while the basal layer of the epidermis, which contains the most undifferentiated cells, is more analogous to the basal epithelium of the limbus [28-30].

The down-regulation of Notch during proliferative states was further demonstrated in the developing mouse cornea as well as the mouse models of corneal wound healing and TPA-induced proliferation. In these latter models, the rate of proliferation peaks in the first 24 h [13,31]. Our results indicate a clear down-regulation of Notch signaling at 24 h when the corneal epithelial cells are most actively proliferating and their differentiation program is temporarily suspended. After 24 h, the level of Notch is quickly restored as the demand for proliferation is reduced and the need arises to re-establish the differentiation program.

The in vitro studies further confirm the in vivo observations. Specifically, Notch activity as measured by the levels of *Hes1* is increased in culture conditions that limit proliferation and promote differentiation. Notch is suppressed during hyperproliferative states when growth and proliferation is more important than differentiation. These results are also consistent with the findings of Thomas et al. who found Notch expression to be absent in the actively dividing corneal epithelial cells in culture. On the other hand, exogenous Notch activation or inhibition in human corneal epithelial cells appears to have the opposite effect. A study by Ma et al. found that Notch inhibition by the γ-secretase inhibitor, L-685458, reduced proliferation and promoted differentiation while Notch activation by soluble Jagged1 increased proliferation and inhibited differentiation in corneal epithelial cell cultures [27]. The reason for these seemingly contradictory results may due in part to the non-Notch mediated effects of γ-secretase inhibitor as well as the dose dependant effects of Notch. In particular, previous studies in neuronal cell cultures have shown that high and low levels of Notch activation actually have the opposite effects [32]. A similar phenomenon is seen in other signaling systems such as TGF-β where high and low levels of the cytokine have the opposite effect on cell proliferation [33]. Current studies are underway to determine the in vivo effects of exogenous Notch activation and inhibition in the mouse corneal epithelium.

In summary, we have demonstrated that Notch signaling is active in the basal and suprabasal corneal epithelial cells. Notch activity is inversely proportional to the degree of proliferation, confirming its role in controlling proliferation and promoting differentiation. In the epidermis, studies have shown that it may be possible to modify the Notch activity to inhibit differentiation and expand the stem cell population [18]. Based on the similarity of the corneal epithelium with the epidermis, Notch signaling may also provide a potential therapeutic pathway by which corneal epithelial proliferation and differentiation can be manipulated. This may be valuable for in vitro applications such as culturing epithelial cells for clinical use and for in vivo conditions such as partial limbal stem cell deficiency.
